# Development of a new promoter to avoid the silencing of genes in the production of recombinant antibodies in chinese hamster ovary cells

**DOI:** 10.1186/s13036-019-0187-y

**Published:** 2019-06-28

**Authors:** Roberto A Zúñiga, Matías Gutiérrez-González, Norberto Collazo, Pablo Hérnan Sotelo, Carolina H Ribeiro, Claudia Altamirano, Carmen Lorenzo, Juan Carlos Aguillón, María Carmen Molina

**Affiliations:** 1 Centro de InmunoBiotecnología, Programa de Inmunología, Instituto de Ciencias Biomédicas (ICBM), Facultad de Medicina, Universidad de Chile, Santiago, Chile; 2 Doctorado en Química, Universidad República Oriental del Uruguay, Montevideo, Uruguay; 3 Departamento de Biotecnología, Facultad de Ciencias Químicas, Universidad Nacional de Asunción, San Lorenzo, Paraguay; 4 Facultad de Química, Universidad República Oriental del Uruguay, Montevideo, Uruguay; 5 Escuela de Ingeniería Bioquímica, Pontificia Universidad Católica de Valparaíso, Valparaíso, Chile; 6 Business Development Department, Fundación Fraunhofer Chile Research, Santiago, Chile; 7 Programa de Doctorado en Farmacología, Facultad de Ciencias Químicas y Farmacéuticas, Universidad de Chile, Santiago, Chile

**Keywords:** Recombinant antibody production, Promoter, Gene expression, Chinese hamster ovary (CHO) cells

## Abstract

**Background:**

The production of recombinant proteins in mammalian cell lines is one of the most important areas in biopharmaceutical industry. Viral transcriptional promoters are widely used to express recombinant proteins in mammalian cell lines. However, these promoters are susceptible to silencing, thus limiting protein productivity. Some CpG islands can avoid the silencing of housekeeping genes; for that reason, they have been used to increase the production of recombinant genes in cells of animal origin. In this study, we evaluated the CpG island of the promoter region of the β-actin gene of *Cricetulus griseous* (Chinese hamster), associated to the Cytomegalovirus (CMV) promoter, to increase recombinant antibodies production in Chinese Hamster Ovary (CHO) cells.

**Results:**

We focused on the non-coding region of CpG island, which we called RegCG. RegCG behaved as a promoter, whose transcriptional activity was mainly commanded by the CAAT and CArG boxes of the proximal promoter. However, the transcription started mainly at the intronic region before the proximal transcription start site. While the CMV promoter was initially more powerful than RegCG, the latter promoter was more resistant to silencing than the CMV promoter in stable cell lines, and its activity was improved when combined with the CMV promoter. Thereby, the chimeric promoter was able to maintain the expression of recombinant antibodies in stable clones for 40 days at an average level 4 times higher than the CMV promoter. Finally, the chimeric promoter showed compatibility with a genetic amplification system by induction with methotrexate in cells deficient in the *dihydrofolate reductase* gene.

**Conclusions:**

We have generated an efficient synthetic hybrid transcription promoter through the combination of RegCG with CMV, which, in stable cell lines, shows greater activity than when both promoters are used separately. Our chimeric promoter is compatible with a genetic amplification system in CHO DG44 cells and makes possible the generation of stable cell lines with high production of recombinant antibodies. We propose that this promoter can be a good alternative for the generation of clones expressing high amount of recombinant proteins, essential for industrial applications.

**Electronic supplementary material:**

The online version of this article (10.1186/s13036-019-0187-y) contains supplementary material, which is available to authorized users.

## Background

The production of recombinant proteins is one of the most important areas in biopharmaceutical industry. Among these proteins, recombinant monoclonal antibodies are of great interest because of their diversity and high specificity [1]; moreover, their therapeutic use has provided a great impact on the treatment of several diseases [2–4].

Chinese Hamster Ovary (CHO) cells have been used as the main platform for the industrial production of antibodies and other complex biopharmaceuticals, as they generate recombinant proteins with correct assembly and glycosylation patterns compatible with their clinical use. Viral transcriptional promoters, such as Cytomegalovirus (CMV) and simian virus 40 (SV40), among others, are strong promoters that are widely used to express recombinant proteins in mammalian cell lines [5]. However, these promoters are susceptible to silencing [6], which is characterized by methylation of transfected DNA in cytosine residues, resulting in the decrease of recombinant protein production [7, 8]. Therefore, obtaining stable cell lines with high levels of gene expression by using standard transfection techniques is a fortuitous event of low frequency. In this sense, ensuring the production of an industrial quality clone requires the isolation and analysis of a large number of clones, which increases development costs.

The CpG islands, which correspond to regions of DNA rich in CpG dinucleotides, provide the maintenance of extensively demethylated regions of the genome. The demethylated state of these islands allows the nucleosome to acquire a less compact configuration, which makes it more accessible to the transcriptional machinery [9–12]. More than 80% of housekeeping (HK) gene promoters are associated with CpG islands [13]. Of note, as HK genes are involved in the basic maintenance of cells, they remain at constant expression levels in all cells [14]. The *β-actin* (*ACTB*) gene is a well-studied HK gene. It has a promoter with a CpG island that extends the proximal transcription start site (TSS), the intron I, and part of the exon II [15]. Such characteristics have led to the use of *ACTB* gene promoters from human [16], chicken [17], shrimp [18], fish [19, 20], and amphioxus [21] origin for the expression of recombinant genes.

The general aim of this work was to evaluate the use of the CpG island of the *ACTB* gene promoter of *Cricetulus griseus* (Chinese hamster) to increase the expression of recombinant antibodies in CHO cells. We studied the promoter characteristics of the *ACTB* gene upon incorporation of a CpG island (RegCG). In addition, we generated chimeric promoters that combined RegCG with the CMV promoter and other regulation elements, such as glucocorticoid response elements (GRE) and their promoter activities were compared with those of CMV promoter alone. Finally, we tested the ability of one chimeric promoter to produce antibodies in CHO cell lines.

## Materials and methods

### DNA isolation and PCR

The plasmids were purified from transformed *E. coli* DH5α cells using the Wizard® Plus SV Minipreps DNA Purification System Kit (Promega, USA), and then cut with restriction enzymes from Invitrogen (USA) or New England Biolabs (USA). The DNA fragments were purified with the Wizard® SV Gel and PCR Clean-Up System Kit (Promega, USA) from agarose gels between 1 to 1.2% (w/v) in TBE buffer. Genomic DNA obtained from CHO-K1 (ATCC® CCL-61™) cells was purified using the PureLink™ Genomic DNA Mini Kit (Invitrogen, USA). The PCR reactions for DNA fragments analysis were carried out with the GoTaq® Green Master Mix Kit (Promega, USA). The PCR reactions to generate cloning fragments were performed with KAPA HiFi HotStart high-fidelity DNA polymerase (Kapa Biosystems, USA).

### Cell culture and media

CHO-K1 cells were cultured in adherent conditions in the chemically defined medium DMEM/F12 (Gibco, USA), supplemented with 5% (v/v) of fetal bovine serum (FBS) (HyClone, USA) and 100 mM of L-glutamine (Gibco, USA). Suspension-adapted, dihydrofolate reductase (DHFR)-deficient CHO DG44 cells were obtained from ThermoFisher Scientific (USA) and initially cultured in a chemically defined CD-DG44 medium (Gibco, USA) with a mixture of sodium hypoxanthine and thymidine (HT).

### Plasmid design and construction

**Fig. 1:**
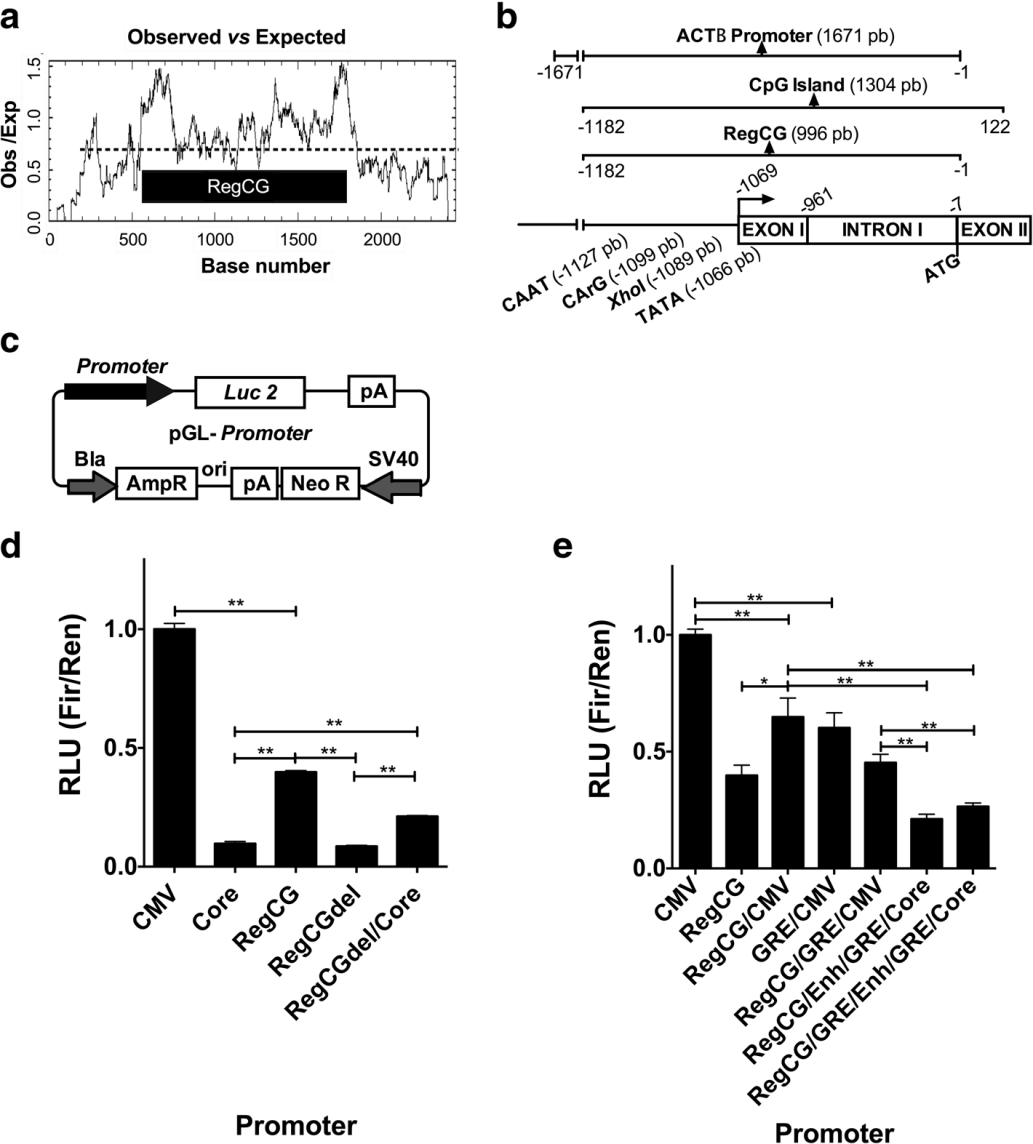
RegCG shows transcriptional activity, which can be improved by the cytomegalovirus (CMV) promoter. **a** Plot of observed versus expected CpG values (Obs / Exp), calculated by the Gardiner-Garden and Frommer method [15], using the Cpgplot tool from the European Bioinformatics Institute (https://www.ebi.ac.uk/Tools/seqstats/emboss_cpgplot/). The RegCG region is indicated in the inside box. **b** Schematic representation of the *ACTB* gene region that contains a CpG island where the promoter, the CpG island, and the region—named RegCG—are indicated. In addition, the CAAT (-1127 pb), CArG (-1099 pb) and TATA (-1066 pb) boxes, the XhoI (-1089 pb) restriction site, the hypothetical proximal TSS, the exon I, the intron I, part of the exon II, and the start codon of the β-actin coding sequence (ATG) are designated. **c** Schematic representation pGL-promoter reporter vectors derived from pGL4.17 vector containing recombinant promoters. **d**, and **e** Transient reporter transcriptional activity of CHO-K1 cells co-transfected with the pGL-promoter vectors which express the firefly luciferase gene, and the normalization pGL4.73 vector, which expresses the renilla luciferase gene. Each promoter is indicated on the x-axis of the graph. Each point represents the activity value, expressed as relative light units (RLU), normalized against renilla activity (Fir/Ren). The RLU measurements were normalized with respect to the average activity of the CMV promoter, which was assigned to a RLU value equal to 1.0. Graphs **d**) are representative of two experiments with six points per measurement in each one. Graph **e**) represents the integration of two experiments with three points per measurement in each one normalized through CMV average activity (*n* = 6). Data are presented as mean values ± standard deviation (S.D.). The one-tailed unpaired t-test, Mann-Whitney test, was used for statistical analysis. * *p* < 0.05 and ** *p* < 0.01

The RegCG fragment was obtained from CHO-K1 cell genomic DNA by PCR using the PactFw and PactRv primers (Additional file 1: Table S1). The CMV promoter and CMV enhancer were obtained by PCR from the pcDNA3.1 (-) vector (Invitrogen, USA) using the CMVcFor01 and CMVrev01 primers to CMV promoter, and CMVcFor01 and ENHVrev01 primers to CMV enhancer (Additional file 1: Table S1). The ENHVrev01 primer anneals immediately upstream the Core region. The ends of the oligonucleotides used for amplifications contained the restriction sites required for insertion in the corresponding vectors (Additional file 1: Table S1). To generate Core and GRE fragment pairs of oligonucleotides of complementary sequences, an amount of 38 to 60 nucleotides (Additional file 1: Table S1 and Additional file 2: Figure S1) that covered the sequences of Core and GRE were synthesized. Each pair of oligonucleotides were hybridized leaving five nucleotides out of phase to generate cohesive ends. Transcription reporter vectors were constructed by inserting the promoter sequences into the polylinker site of pGL4.17 vector (Promega, USA) (Fig. 1c). The bicistronic vectors for the expression of an anti-human CD20 antibody were constructed by modifying a specific reporter vector where the luciferase gene was replaced by an insert containing the genes of the light and heavy chains of the antibody separated by an internal ribosome entry site (IRES) sequence (Fig. 4a). In order to express heavy and light chain genes of an anti-human tumor necrosis factor (TNF) antibody, two vectors were constructed. Briefly, the vector for the heavy chain (TNF-H) expression was constructed based on the reporter vector pGL-RegCG/GRE/CMV, in which the Luc gene was replaced by the heavy chain coding gene of the anti-TNF antibody (pRegCG/GRE/CMV-TNF-H). The vector for the light chain (TNF-L) expression was constructed by replacing the CMV promoter of the pOptiVEC™-TOPO® vector (Invitrogen, USA), between the restriction sites Sal I and BamHI, by a fragment containing the RegCG/GRE/CMC vector, followed by the light coding chain gene of the anti-TNF antibody. This fragment was inserted upstream IRES, allowing the co-expression of the anti-*TNF-L* and *dhfr* genes under the control of the same promoter (pRegCG/GRE/CMV-TNF-L) (Fig. 5a).

### Transfection of adherent CHO-K1 cells

For transient assays to generate stable cells lines, the following transfection protocol was carried out: the parental CHO-K1 cells were incubated at 300,000 cells/mL in 500 μL of DMEM-F12 medium (Gibco, USA) supplemented with 10% FBS and 100 mM L-glutamine at 37 °C and 5% (v/v) CO_2_. When reaching 80 to 90% of confluence, the cells were transfected; for this purpose, the culture medium was removed and a mixture containing 500 ng of expression vector was added with 20 μL of Lipofectamine 2000 CD (Invitrogen, USA) in 100 μL of OptiMEM medium (Invitrogen, USA), and cells were incubated for 4 h at 37 °C and 5% CO_2_. Next, the transfection mixture was removed, cells were fed with 500 μL of DMEM-F12 medium supplemented with 10% FBS and then cells were incubated for 48 h at 37 °C and 5% CO_2_.

### Transcriptional reporter assays

To measure the transient activity of promoters, CHO-K1 cells were co-transfected with the transcription reporter vector derived from the pGL4.17 vector (Promega, USA), and the normalizing pGL4.73 vector (Promega, USA), following the protocol described above. The transfected cells were collected and lysed after 48 h of culture, according to the instructions of the “Dual-Glo® Luciferase Assay System” (Promega, USA), and luminescence was measured in a Luminoskan ASCENT luminometer (Thermo Electronic Corporation). The promoter activity was calculated by the quotient between the luciferase activities of firefly and renilla (FIR/REN) and expressed as relative light units (RLU).

To measure the activity of promoters in stable cell lines, CHO-K1 cells were transfected with pre-linearized expression reporter vectors followed by clone isolation through limiting dilution in the presence of the selection antibiotic G418 (Gibco, USA); dilutions were performed in 96-well culture plates with DMEM-F12 medium supplemented with 10% FBS, and 100 mM L-glutamine at 37 °C and 5% CO_2_, in the presence of the selection antibiotic G418. Reporter activity from the cell lysate was determined using the Bright-Glo™ luciferase assay system (Promega, USA), and normalized by the total protein concentration determined by the Bradford assay. Reporter activity was expressed as RLU, equivalent to the quotient between the luciferase activity of firefly and the total protein concentration (FIR/Prot).

### Generation of anti-human CD20 antibody-producing cell lines

CHO-K1 cells were transfected with a pre-linearized bicistronic expression vector (pRegCG/GRE/CMV-aCD20LH) coding for the heavy and light chains of the anti-human CD20 antibody in one vector, according to the protocol described above.

### Generation of anti-human TNF antibody-producing cell lines

The parental CHO DG44 cells were co-transfected with the linearized plasmids pRegCG/GRE/CMV-aTNF-H and pRegCG/GRE/CMV-aTNF-L, which code for the heavy and light chains of the chimeric anti-human TNF antibody, respectively, as described in Torres et al., 2018 [22]. The cells were transfected using the FreeStyle™ MAX System Reagent (Invitrogen, USA), following the manufacturer’s instructions. To obtain clones of higher production, the transfected cells were subjected to gene amplification by the methotrexate (MTX)-based method and enriched by flow cytometry at 4 °C, as described in Torres et al. (2018) [22]. Finally, clonally-derived cell lines were isolated in semi-solid ClonaCell™ medium (STEMCELL Technologies Inc., Canada) [22] and cultured for 15 days; visible colonies were then isolated manually by aspiration with a micropipette. Approximately 600 clones were isolated and grown in 96-well culture plates in suspension. The clones were evaluated in terms of production of the anti-TNF antibody by enzyme-linked immunosorbent assay (ELISA), as described below. In each step of scaling, the 10 clones of higher production were selected for their relative level of antibody production. Next, cell lines were scaled sequentially until reaching cultures in Erlenmeyers of 125 mL. Finally, antibody-producing cultures were established by growing the cells at 37 °C in a chemically defined BalanCD®CHO Growth A medium (Irvine Scientific, USA) supplemented with 4 mM L-Glutamine. Cells were cultured in duplicate in 125 mL Spinner flasks (TechneTM, UK) with a working volume of 60 mL, seeded at 0.8 × 10^6^ cells/mL, and ≥ 95% viability. All cell cultures were incubated in a Mco-17Aic CO_2_ Incubator (SANYO Electric Co., Ltd., Japan) at a 5% CO_2_ enriched atmosphere.

### Determination of antibody production

The production levels of anti-human CD20 and anti-human TNF antibodies in CHO-K1 and CHO DG44 cells were determined by measuring the concentration of the antibodies in the culture supernatants, which was quantified by an antigen capture ELISA prepared in our laboratory, and described in [22].

### Gene expression analysis by RT-PCR

RNA was extracted from cells using E.Z.N.A.® Total RNA Kit I (Omega Bio-tek Inc., USA), according to the manufacturer’s instructions. RNA concentration was quantified using a Synergy 2 Spectrophotometer (BIO-TEK Instruments, Inc., USA). RNA extracts (2 μg) were treated with DNase I (Thermo Fisher Scientific, USA) to remove any trace of genomic DNA contamination. Reverse transcriptase production of cDNA from the RNA was performed using an Affinity Script enzyme (Agilent Genomics, USA) and oligo dT. The amplification of the specific cDNA was carried out by PCR with the GoTaq® Green Master Mix Kit (Promega, USA), and the amplification products were analyzed by electrophoresis on 1% (w/v) agarose gels.

## Results

In order to identify the CpG island associated with the *ACTB* gene of *Cricetulus griceus,* we analyzed the *ACTB* gene promoter sequence obtained from a contig of the genome of *Cricetulus griseus* using nBLAST of National Center for Biotechnology Information (NCBI). The sequence of this contig was compared with its orthologous human, mouse, and rat genomic DNA sequences, using Clustal Omega (European Bioinformatics Institute, EMBL-EBI), to identify the promoter and coding regions (Fig. 1a). The sequence was numbered using as reference (+ 1) to the first base of the start codon of the *ACTB* gene. We identified phylogenetically conserved elements of *ACTB* gene promoters in eukaryotes [23, 24], such as CAAT (-1126 to -1123), CArG (-1098 to -1089) and TATA boxes (-1066 to -1063), and a proximal TSS (-1069), which starts the exon I, followed by the intron I in the non-coding region (-1070 to -7), along with the exon II, which starts at -6 (Fig. 1b). In this contig, using the EMBOSS Cpgplot tools (European Bioinformatics Institute, EMBL-EBI), we identified a CpG island with a 67.7% CG content of 1304 bp that starts at position -1182 of the 5’UTR region of the *ACTB* gene promoter Fig. 1a). This CpG island did not include the 5’ region of the promoter; however, it included the proximal promoter elements (CAAT, CArG and TATA boxes), exon I, and intron I, and ended at the position + 122, falling in exon II. Therefore, it also included a small portion of the coding region of the *ACTB* gene (Fig. 1b).

### The CpG island of the *ACTB* gene has transcriptional activity, which is enhanced by the CMV promoter

Since the CpG island included the characteristic elements of the *ACTB* gene promoter, we decided to test whether this promoter had transcriptional activity. For that purpose, we cloned the non-coding CpG island region (RegCG) into the transcription reporter vector pGL4.17 (Fig. 1c). To avoid translation from the start codon of the *ACTB* gene above the start codon of the recombinant gene, the 122 bp of the coding region of the *ACTB* gene was not included. The activity of this region was compared with the activity of the CMV promoter and a short CMV promoter containing only the CAAT and TATA boxes motifs (Core) through transient expression assays performed on the CHO-K1 cell line after 48 h of culture. We observed that the RegCG promoter possessed approximately 40% activity from the CMV promoter and that its activity was 4-fold greater than that of the CMV Core promoter (Fig. 1d). Therefore, these results indicated that RegCG could act as a promoter.

Since RegCG does not contain the 5’ region of the *ACTB* gene promoter, the role of the CArG and CAAT elements in the transcriptional activity of RegCG was analyzed. For this purpose, we built a CpG mutant that did not contain the CAAT and CArG boxes, and the sequence located upstream the *Xho*I restriction site was eliminated (RegCGdel). This mutant showed a 5-fold decrease in activity compared to the RegCG promoter (Fig. 1d), suggesting that CAAT and CArG elements are critical for the promoter activity. However, the inactivation of the mutant was not absolute, since it showed some transcriptional activity. The activity of this mutant was comparable to that of the CMV Core promoter, and it was capable of increasing the activity of the CMV Core promoter (RegCGdel/Core) (Fig. 1d). The activity of the mutant promoter may have been mediated by the TATA box motif.

In order to increase RegCG promoter activity, we generated the chimeric RegCG/CMV promoter by inserting RegCG upstream the CMV promoter, followed by its cloning into the pGL4.17 vector. The analysis of the activity of the RegCG/CMV promoter, measured in transient transfection assays, showed that this association allowed the chimeric promoter to achieve reporter activity levels 25% higher than the RegCG promoter, which suggests that CMV adds its activity to RegCG (Fig. 1e). On the other hand, the chimeric promoter presented less activity than the CMV promoter, reaching only 64.9% activity (Fig. 1e), suggesting that RegCG interferes with the CMV promoter activity.

Additionally, we incorporated a tandem of five GRE upstream the CMV promoter, which was useful to test the effect of the presence of additional elements in the chimeric promoter activity. This element was inserted upstream the CMV promoter, generating the GRE/CMV and RegCG/GRE/CMV promoters, and inside the CMV promoter, between the enhancer and the Core regions, generating the RegCG/Enh/GRE/Core and RegCG/GRE/Enh/GRE/Core promoters. The transcriptional activity in transient expression assays showed that the GRE element located upstream the CMV promoter caused a decreased activity with respect to the activity of the CMV promoter (Fig. 1e), as observed by the addition of RegCG. Moreover, introduction of GRE inside the CMV promoter generated a more drastic decrease in the transcriptional activity. Therefore, the incorporation of additional elements in the chimeric promoter decreases its activity, which may reach even less activity when compared to that of the RegCG promoter alone. Thus, in transient assays, the CMV promoter is the best promoter among the variants tested.

### Promoters that contain RegCG are more resistant to silencing

**Fig. 2:**
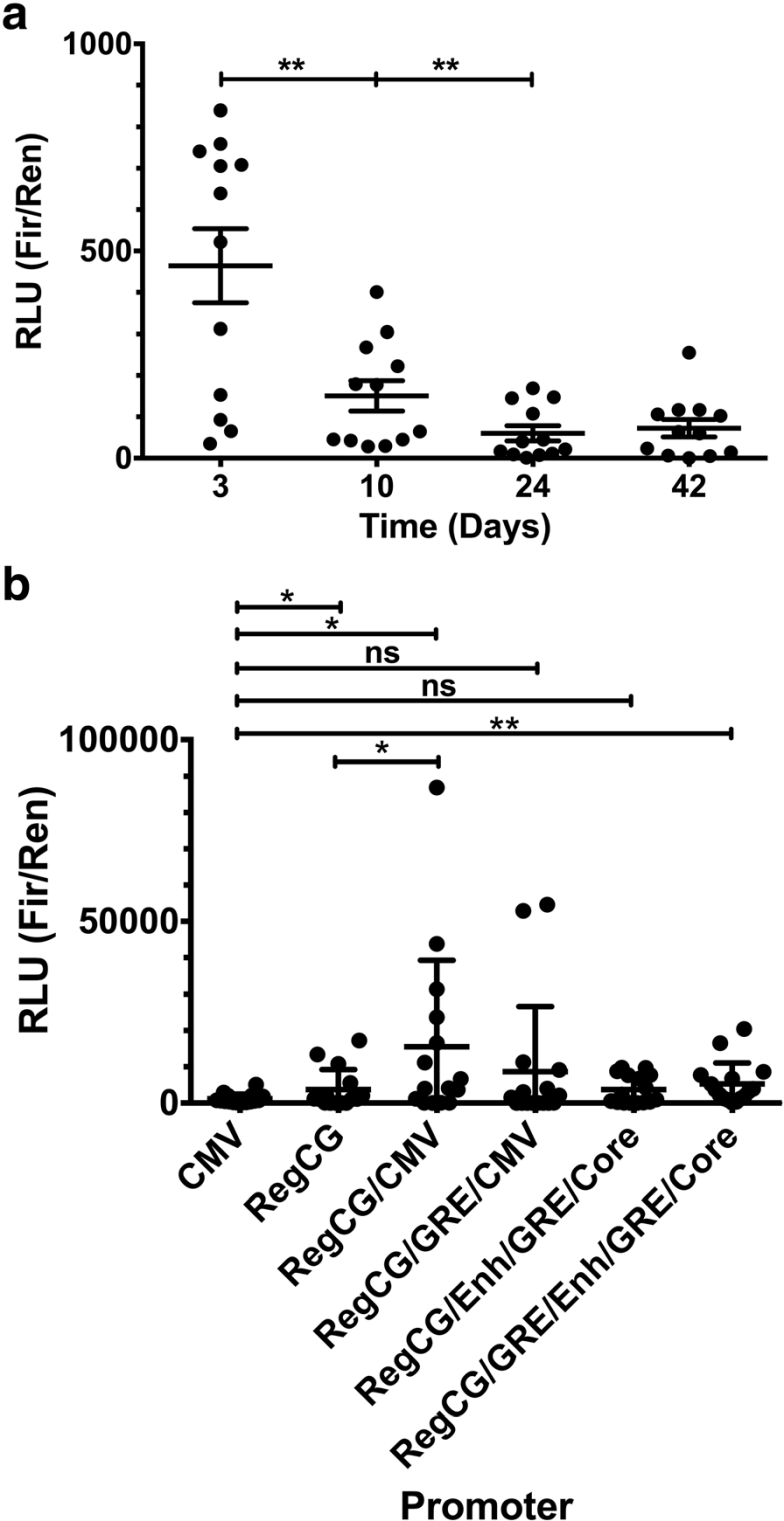
Promoters containing RegCG are resistant to silencing in stable cell lines. Transcriptional activity in stable lines of CHO-K1 cells transfected with the reporter vectors and cloned by limiting dilution. The activity of the promoters was expressed in relative light units (RLU) calculated as the ratio between the luminescence and the concentration of total proteins in the cell lysates (Fir/Ren). In **a**) the decay of the activity of the cytomegalovirus (CMV) promoter in a group of clones is shown up to 41 days after the removal of the selection antibiotic (*n* = 12). In **b**) the activity of the promoters pGL-CMV, pGL-RegCG, pGL-RegCG/CMV, pGL-RegCG/GRE/CMV, pGL-RegCG/Enh/GRE/Core, and pGL-RegCG/GRE/Enh/GRE/Core of a group of randomly chosen clones on day 39 after selection was withdrawn (*n* = 16). Data are presented as mean values ± S.D. The one-tailed unpaired t-test, Mann-Whitney test, was used for statistical analysis. * p < 0.01 and ** p < 0.05

Promoter silencing leads to a decrease in their transcriptional activity. To test the effect of RegCG on the silencing of the CMV promoter, the activity of RegCG/CMV, RegCG/GRE/CMV, RegCG/Enh/GRE/Core, and RegCG/GRE/Enh/GRE/Core promoters was evaluated by a reporter system of the transcription in stable cell lines, and compared with the activity of CMV and RegCG promoters alone. Stable CHO-K1 cell lines were generated and cultured in the presence of the selection antibiotic until the culture was established. After that, the selector was removed to freely allow the silencing of the recombinant gene. Analysis of the activity decay of the CMV promoter revealed that, at 40 days of culture, and after the withdrawal of the selection antibiotic, there was a stabilized level of promoter activity, reaching about 10% of the initial values (Fig. 2a). To determine whether the different promoters presented better resistance to silencing than the CMV promoter, 16 clones of each transfection were selected at random and sub-cultured until day 39 after removal of the selection antibiotic. At this time point, the reporter activity of each clone was analyzed and compared with clones bearing-the CMV promoter (Fig. 2b). The results showed that the clones carrying the RegCG promoter had a 3-fold increase in average activity compared with clones carrying the CMV promoter, indicating that RegCG induces resistance to gene silencing. As expected, clones with RegCG/CMV exceeded 5-fold the average activity of clones carrying the RegCG promoter, suggesting that there is an additive effect between RegCG and the CMV promoter. The second most active promoter was RegCG/GRE/CMV, whose clones reached a 2-fold increase in the activity compared to RegCG clones.

To test the role of RegCG in the RegCG/CMV and RegCG/GRE/CMV promoters in stabilized cell lines, we used chimeric promoters with disrupted CMV promoter by the insertion of a GRE element between its enhancer and Core regions (RegCG/Enh/GRE/Core and RegCG/GRE/Enh/GRE/Core). Although promoters with the disrupted CMV promoter had less transcriptional activity than RegCG in transient assays (Fig. 1d), surprisingly, in stable clones, these promoters showed similar activities with respect to RegCG (Fig. 2b). Therefore, RegCG maintains the transcriptional activity of the chimeric promoters when the activity of the CMV promoter is decreased. On the other hand, these data also showed that the presence of the CMV promoter is important for the increased activity of the chimeric promoter. Altogether, these results suggest that RegCG and CMV promoters may function in tandem, resulting in an additive effect that is reflected in increased recombinant protein production. However, given that in the individual analysis of the RegCG and CMV promoters it was observed that RegCG maintains higher levels of transcription in stabilized clones than the CMV promoter, we conclude that RegCG has an active role in decreasing the silencing of the chimeric promoters.

### The transcriptional activity of RegCG starts in an intronic TSS

**Fig. 3:**
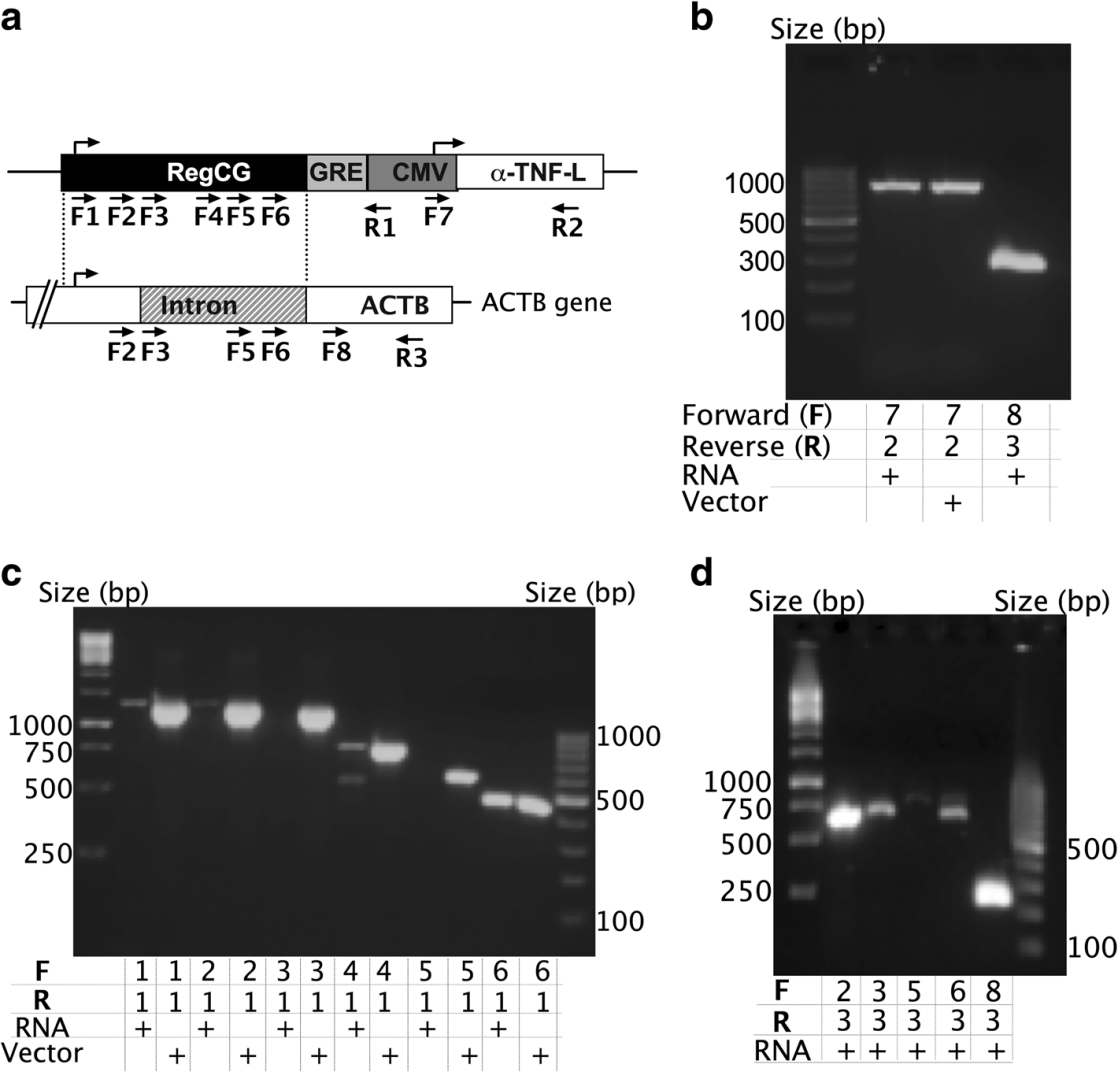
Promoter RegCG has a transcription start site in the intron I. The transcription start site (TSS) from RegCG in the chimeric promoter RegCG/GRE/CMV was analyzed in cells derived from CHO DG44 cells co-transfected with pRegCG/GRE/CMV-aTNF-L and pRegCG/GRE/CMV-aTNF-H vectors. Total RNA was isolated from a culture of cells in the exponential phase, and cDNA was prepared. Primers that anneal to different parts of RegCG and cytomegalovirus (CMV) were used. **a**) Schematic representation of the recombinant anti-TNF-L gene commanded by the RegCG/GRE/CMV promoter, and the endogenous *ACTB* gene. The dotted lines indicate the region of the corresponding β-actin gene with the RegCG portion of the recombinant promoter. The lower arrows indicate the position and direction of the primers, where F and R indicate the forward and reverse primers, respectively. The upper arrows indicate the position of the theoretical TSS. **b** and **c** RT-PCR analysis of the recombinant promoter RegCG/GRE/CMV. At the bottom of the figure, RNA (total RNA), vector (plasmid DNA of pRegCG/GRE/CMV-aTNF-L vector), and primers are indicated. The primers used are indicated with numbers corresponding with numbering in A. This assay is representative of two experiments. **d** RT-PCR analysis of the *β-actin* gene

In order to determine whether the RegCG region of the RegCG/GRE/CMV promoter was transcriptionally active, CHO DG44 cell line, transfected with vectors that code for an anti-human TNF antibody (Fig. 5a), were harvested in the exponential phase of a suspension culture and specific RT-PCR assays were performed. Several forward (F) primers were designed to recognize the RegCG region of the chimeric promoter: primers F1 and F2 for exon I; primer F3 for the intersection exon/intron; and primers F4, F5 and F6 for intron I (Fig. 3a). To avoid the amplification of the endogenous promoter of the β-actin gene, we used a reverse (R) primer to anneal the enhancer of the CMV promoter (primer R1). The absence of genomic DNA was confirmed by the size of the amplification product with primers F8 and R3 (Fig. 3b, line F8/R3), which flank an intron of the *ACTB* gene with an expected amplification size of 711 bp from genomic DNA and 256 bp from mRNA. The intensity of amplification from recombinant mRNA was comparable to amplification from plasmid DNA vector (Fig. 3b, lines F7/R2).

In these tests, amplification was observed with F1, F2, F4 and F6 primers in the sample with mRNA (Fig. 3c), showing that the RegCG portion of the chimeric promoter presents transcriptional activity. In addition, we observed that the size of the amplification products obtained with F1 and F2 primers annealed upstream the intron I, and that F4 and F6 primers annealed within the intron I, and are of the same size as those obtained with their vector DNA controls (Fig. 3c, lanes F1/R1, F2/R1, F4/R1 and F6/R1), which showed that the transcript produced is not processed. When the intensities of the amplifications were compared with the mRNA sample with respect to its vector DNA control, it was observed that amplification with the F6 primer was greater than that obtained with the primers that annealed upstream (Fig. 3c, lanes F6/R1). This difference in intensity suggests that there is a possible start site for additional transcription within the intron that would be located between the annealing sites of F5 and F6 primers.

Using the same cDNA preparation, we tested the functionality of primary TSS of the endogenous *ACTB* gene, as well as some of the forward primers used in the analysis of transcription from the chimeric promoter. In order to achieve a specific amplification, we used a reverse primer that recognizes the coding region of the *ACTB* gene. In this assay, using the F2 primer, we found a potent amplification downstream the theoretical proximal TSS of the *ACTB* gene (Fig. 3d, F2/R3), which corresponds to the expected amplification product after splicing of the messenger of 744 pb (Fig. 3d, F8/R3). The amplification products obtained with F3, F5 and F6 primers showed amplification sizes that did not coincide with those expected for unprocessed RNA (Fig. 3d, F6/R3). These results indicate that RegCG has a different behavior than the *ACTB* promoter, where, in the RegCG region of the chimeric promoter, the main responsible for the transcriptional activity is the intronic TSS, unlike the endogenous promoter, where the main responsible for the transcriptional activity is the proximal TSS.

### Promoters containing RegCG increase the production of a recombinant antibody

**Fig. 4:**
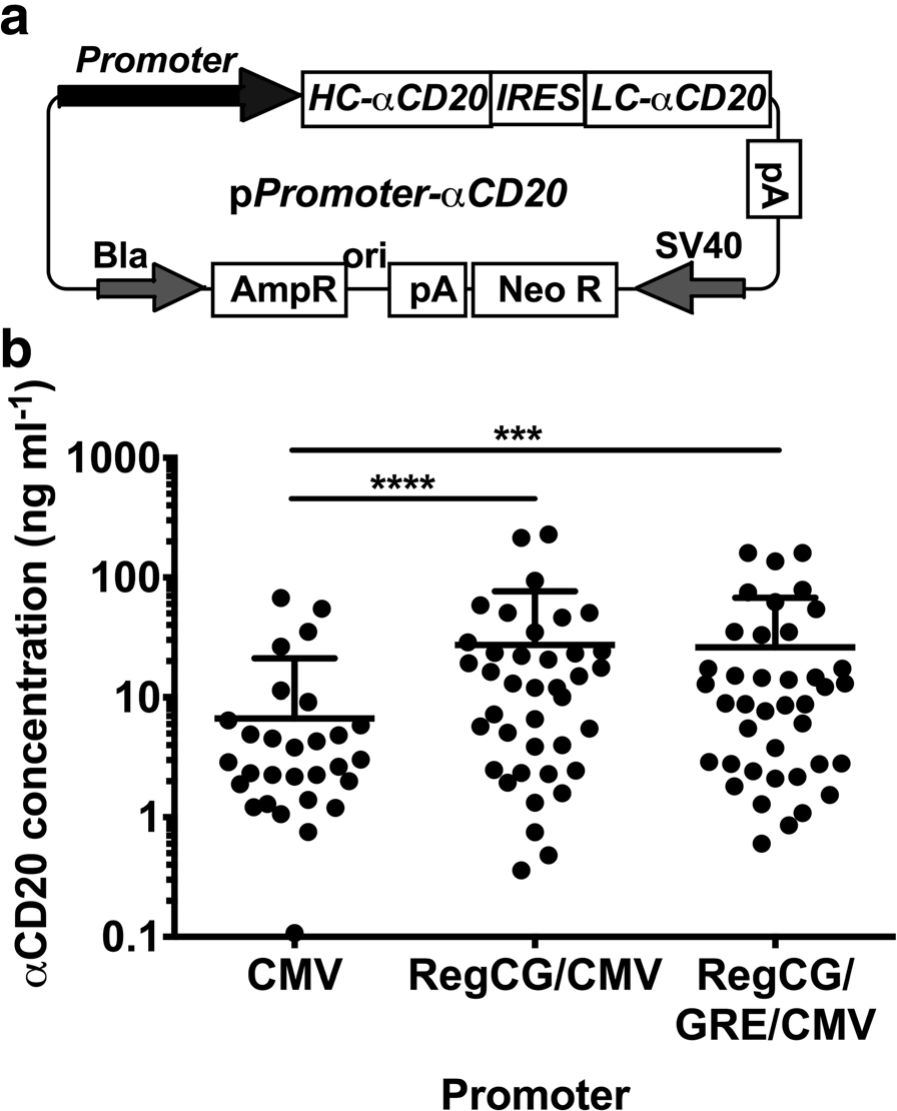
Promoters containing RegCG increase the production levels of an anti human-CD20 antibody in CHO-K1 cells. CHO-K1 cells transfected with pRegCG/GRE/CMV-aCD20, pRegCG/CMV-aCD20 and pCMV-aCD20 vectors and cloned by limiting dilution were cultured for 35 days after removal of the selection antibiotic; supernatants were obtained and the volumetric production of antibodies was measured by ELISA. **a** Schematic representation of recombinant promoters cloned in biscistronic vectors to express a human anti-CD20 antibody. **b** Antibody concentration from 39 clones with three different promoters: CMV: 6.2 ng/mL, RegCG/CMV: 25.3 ng/mL and RegCG/GRE/CMV: 24.3 ng/mL (horizontal bar indicates standard error of the mean, (SEM)). Data were analyzed by the Kruskal-Wallis test (ANOVA), followed by the Dunn test. *** *p* < 0.001, **** *p* < 0.0001 (*n* = 39)

The effect of RegCG-containing promoters in the production of recombinant antibodies was tested by the expression of an anti-human CD20 chimeric antibody in CHO cells using a bicistronic expression system. The RegCG/CMV and RegCG/GRE/CMV promoters were selected because they showed the highest transcriptional activity with the reporter system in stable clones (Fig. 2b). The bicistronic vectors were constructed by replacing the luciferase gene in the reporter vectors containing the respective promoters with the heavy and light chain genes of the anti-CD20 antibody (anti-CD20-H and anti-CD20-L, respectively) separated by an IRES (Fig. 4a). Stable recombinant CHO-K1 cell lines were generated by transfection with expression vectors RegCG/CMV-aCD20, RegCG/GRE/CMV-aCD20 and CMV-aCD20, which contained RegCG/CMV and RegCG/GRE/CMV chimeric promoters, and the CMV promoter, respectively. Fifty-six clones of each construct were collected randomly and cultured in the absence of selection antibiotics for a period of approximately 40 days to allow promoter silencing. This period of silencing was previously determined for clones generated with the CMV promoter (Fig. 2a). As shown on Fig. 4b, 30, out of 56 CMV clones produced a measurable antibody concentration, whereas for RegCG/CMV and RegCG/GRE/CMV clones, 39 and 40 clones, respectively, produced a measurable antibody concentration. The average production of RegCG/CMV and RegCG/GRE/CMV clones was 4-fold higher than the production of clones bearing-the CMV promoter (Fig. 4b). The same difference was also observed when analyzing the top 5 clones of each construct (data not shown). No statistical differences were found in the average production of antibodies from RegCG/CMV and RegCG/GRE/CMV clones, which indicates that the decrease in the activity of the promoters caused by the insertion of GRE does not drastically affect promoter activity in stable cell lines. Therefore, these results confirm that addition of RegCG to the CMV promoter significantly increases the production of recombinant proteins after a period of stabilization of the recombinant clones.

### Expression of a recombinant antibody under the control of RegCG/GRE/CMV promoter in suspension cultures

**Fig. 5:**
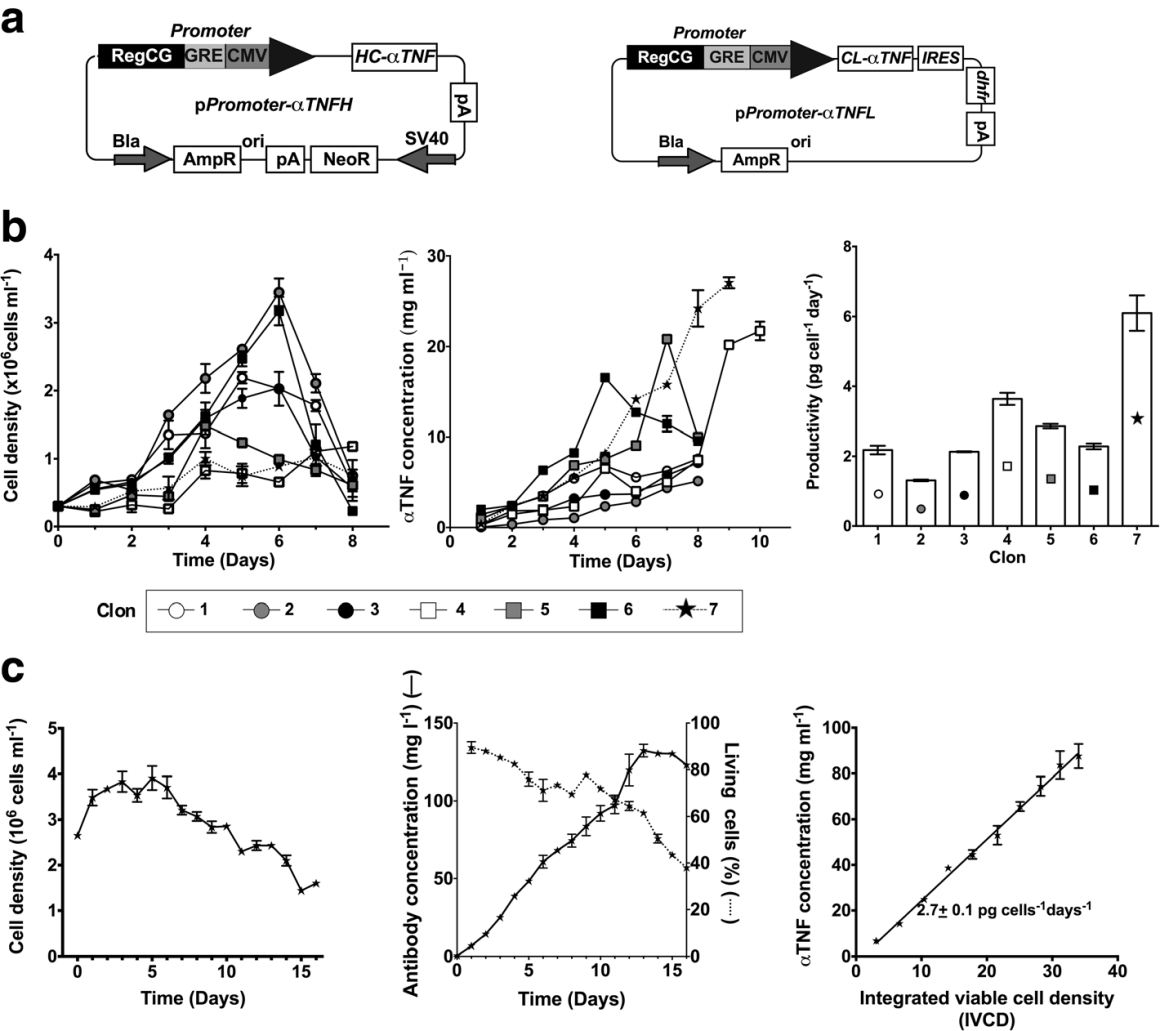
Production of a recombinant antibody in CHO DG44 cells with a promoter containing RegCG. CHO DG44 cells were co-transfected with vectors expressing an anti -human tumor necrosis factor (TNF) antibody under the control of the chimeric promoter RegCG/GRE/CMV, subjected to genetic amplification by methotrexate; cell populations with higher production of antibodies were isolated by FACS. Clones were seeded in semi-solid medium, cultured in 96-well plates and their volumetric antibody production was analyzed by ELISA. **a** Schematic representation of pRegCG/GRE/CMV-aTNF-L and pRegCG/GRE/CMV-aTNF-H vectors to express the light and high chains of a human anti-TNF-L antibody. **b** The volumetric antibody production, viability, and specific productivity of the 7 highest production clones cultured in 125 mL flasks are shown. **c** The clone with the highest production level (clone number 7) was grown in a Spinner bottle. Cell density, viability, volumetric production, and specific productivity is shown. Representative result of three independent experiments

To test the ability of the RegCG/GRE/CMV promoter to generate a clone for industrial production, CHO DG44 cells, deficient in the *dhfr* gene, were co-transfected by lipofection with vectors that express the light and heavy chains of an anti-TNF recombinant antibody, both of them controlled by the RegCG/GRE/CMV promoter (Fig. 5a). The vectors containing the heavy and light chains contained the *dhfr* gene and the neomycin resistance gene (neo®) as the selection systems, and clones were treated with methotrexate. At the end of the selection process, 6 clones were obtained that showed a productivity equal to or greater than 2 pg/cell/day (Fig. 5b). Among these clones, clone 6 was selected and cultured in a Spinner bottle. The maximum antibody production of this clone was 132 mg/L, which was reached at 13 days of culture, when the cells had a viability above 60% (Fig. 5c). Therefore, we were able to select clones with productivity over 2 pg cell^-1^ days^-1^ at a ratio of 1 in 100 clones selected.

## Discussion

The use of CHO cells for therapeutic proteins production has revolutionized the medical research and protein manufacturing fields, since they generate large-scale recombinant proteins with correct assembly and glycosylation patterns compatible with their clinical use. Gene silencing due to transcriptional inactivation by methylation often prevents long-term stability of protein production, which is an inherent drawback for an efficient establishment of stable cell lines with high levels of gene expression [8]. Although the CMV promoter provides high levels of gene expression, several authors have demonstrated the susceptibility of this promoter to gene silencing [6]. Promoters containing CpG islands, which are regions practically devoid of methylation, have been associated with transcription initiation [25]. In the present study, we evaluated the capacity of a predicted CpG island in the promoter of the *ACTB* gene of *Cricetulus griseus* to improve the production of recombinant proteins in CHO cells. This region, which we called RegCG, includes the proximal promoter, the exon I, and the intron I of the *ACTB* gene. We demonstrate that RegCG behaves as a promoter and that it is more resistant to silencing than the CMV promoter.

Several tools have been described to target the human *ACTB* gene promoter; however, the promoter regions analyzed in these studies were different from the one characterized here. For example, Damdindorj et al. described a minimal portion of the human *ACTB* gene promoter that is able to sustain gene expression in stable cell lines. This portion of the promoter includes the 5’ region of the promoter and the proximal promoter, where the CAAT, CArG, and TATA boxes are found 30 bp upstream of the exon I. Nevertheless, this sequence does not include the intron I of the gene [16], which has been shown to correlate positively with gene expression levels [26]. Another example is the cytomegalovirus early enhancer chicken/β-actin (CAG) promoter, a well-known synthetic promoter that provides efficient and stable gene expression in different cell lines. This includes the proximal promoter, the exon I, and a large sequence of the intron I of the chicken *ACTB* gene, followed by the splicing acceptor of the rabbit β-globin gene [27, 28]. In this promoter, the 5’ end of the *ACTB* gene promoter was replaced by the enhancer of the CMV promoter, resulting in prolonged transcriptional activity, which was more stable than the CMV promoter [16]. Here, we have generated the RegCG promoter that is more resistant to silencing than the CMV promoter, which included a fragment of the *Cricetulus griseus ACTB* gene promoter, although our promoter did not include a 5’ end enhancer. Therefore, it is possible that inclusion of a 5’ end enhancer sequence into the RegCG promoter may potentiate gene transcription.

Here we also generated a synthetic chimeric promoter containing RecCG and CMV promoters and provide evidence that this chimeric promoter presents higher activity than the CMV and RegCG promoters alone in stables clones. Our finding is in accordance with the results obtained by William et al., who have previously shown that the incorporation of Ubiquitous Chromatin Opening Elements (UCOE) la methylation free CgG island upstream the CMV promoter decreased silencing of the recombinant gene with the consequent increase in the production of recombinant proteins [29]. This effect is caused by inhibiting the promoter gene silencing, a mechanism well described for the CMV promoter [6]. However, Brendel et al. demonstrated that, although providing protection to the silencing of genes, UCOE does not protect a viral promoter located downstream from it from methylation [30]. While it is possible that RegCG does not protect the CMV promoter from silencing in chimeric promoters, we observed that RegCG/CMV promoter reached is more activity than the CMV promoter alone (Fig. 2b), indicating that this chimeric promoter shows greater activity than the sum of the activities of RegCG and CMV promoters individually. Thus, the contribution of CMV to the activity of the chimeric promoter appears to be greater than that observed for clones that contain the CMV promoter alone after the stabilization period. Whether the silencing mechanisms of CMV in chimeric promoters is dependent on RegCG is an aspect that should be clarified in order to understand the exact mechanism of these promoters.

Feng et al. reported an alternative isoform of an amphiouxus *ACTB* gene of embryonic expression, whose transcription begins in the intron I, which is commanded by its own CAAT, CArG and TATA elements that are present inside the intron I [21]. In mammals, the intron I of the *ACTB* gene also contains a CArG box [23], which may present some regulatory function in the intronic TSS. In contrast, intronic CArG box of the RegCG promoter probably is not regulating the intronic TSS, since this is located downstream the intronic TSS, and contains a T for a C mutation, which affects the consensus sequence (Additional file 3: Figure S2). In addition, we observed a decreased reporter activity in the RegCG mutant (RegCGdel), which lacks the CAAT and CArG boxes of the proximal promoter, indicating that these elements command the activity of RegCG. These results suggest that the intronic TSS is commanded by the CAAT and/or CArG boxes of the proximal promoter and not by intronic regulatory elements.

Recent evidence shows that there is coordination between the transcription machinery, the splicing machinery and the state of chromatin [31, 32]. It has been proposed that the splicing process of some genes increases the transcriptional activity of the promoter [31, 32]. However, the intronic TSS of some genes are activated independently of the splicing process, while it depends on the DNA sequence [33, 34]. Here, the characteristics of the intron sequence of the *ACTB* gene may facilitate a nucleosomal conformation that makes it more accessible to the transcription machinery [33]. Interestingly, these characteristics have also been attributed to the CpG island, where the demethylated promoters have a nucleosome-free region surrounding the TSS. For that reason, chromatin in this area would be intrinsically accessible without the need for a nucleosome shift [33]. Therefore, the initiation of transcription within the RegCG intron supports the idea that its transcriptional activity is due to its CpG island features, independent of the splicing process.

The UCOE has been widely studied for their capacity to improve CMV promoter activity in stably transfected CHO cells [29, 35]. The increased antibody production yield obtained with our chimeric promoters (RegCG/CMV and RegCG/GRE/CMV) compared with CMV promoter was similar to that previously reported by Betts et al, who used a UCOE incorporated upstream the CMV promoter [35]. On the other hand, we incorporated a tandem of 5 GRE between RegCG and CMV (RegCG/GRE/CMV) and tested the induction capacity of this promoter with dexamethasone, although no significant increase in gene expression was observed (data not shown). Nevertheless, the presence of the GRE insert did not affect the antibody production in stable cell lines with the RegCG/GRE/CMV chimeric promoter with respect to RegCG/CMV. Therefore, another change in the chimeric promoter could be used to re-establish the splicing capacity of the intron I present in RegCG to evaluate whether it can improve the activity of the promoter, making it more similar to the CAG promoter [27]. Alternatively, RegCG can be tested alone, or in combination with other promoters (such as SV40 promoter) to improve the production of antibodies.

## Conclusions

We have generated an efficient transcription promoter by combining a CpG island fragment of the *ACTB* gene from *Cricetulus griseus* (RegCG) with the CMV promoter. RegCG behaves as a promoter that sustains the expression of recombinant proteins in stable cell lines, which is more resistant to gene silencing than the CMV promoter. The generation of promoters by the combination of RegCG with CMV results in promoters with greater activity in stable cell lines as compared to both promoters separately. We showed that these chimeric promoters are compatible with the genetic amplification system in CHO DG44 cells and allow the generation of stable cell lines with high production of recombinant antibodies. We propose that this promoter can be used for the generation of industrial cell lines for the production of recombinant antibodies.

## Additional files

Additional file 1: **Table S1.** List of primers. (DOCX 18 kb)

Additional file 2: **Figure S1.** Pairing of the primers for the synthesis of a glucocorticoid response element (GRE) tandem. The alignment of the hybridized primers is shown, prior to their ligation with T4 Ligase through the cohesive ends generated. a) Synthesis of short portion of CMV promoter (Core). b) Synthesis of a tandem of five Glucocorticoid Response Element (GRE). In celestial the palindromic elements of the GRE site are shown, and in yellow the cohesive ends compatible with restriction enzymes are shown. (DOCX 13 kb)

Additional file 3: **Figure S2.** Alignment of promoter sequences of the gene for the beta actin of Gallus (Gall), Human (Hum), Chinese Hamster (CHO), mouse (Mus) and rat (Rat). In Amarillo, the CAAT, TATA and CArG boxes stand out. In green, the *Xho*I restriction site and the start codon of beta-actin are highlighted. The arrows> and < indicate the start and end, respectively of the CpG Island, the start of transcription and the intron I for the sequence of the CHO cell genome. (DOCX 16 kb)

## Data Availability

The data and materials used and/or analyzed during the current study are available from the corresponding author on reasonable request.
