# Supplementary material for: Development of a new promoter to avoid the silencing of genes in the production of recombinant antibodies in chinese hamster ovary cells
Source: J Biol Eng. 2019 Jun 28;13:59. doi: 10.1186/s13036-019-0187-y (PMC6599231; doi:10.1186/s13036-019-0187-y)
Supplement: Supplementary file 1 — Table S1. List of primers. (DOCX 18 kb) [file 13036_2019_187_MOESM1_ESM.docx]

Tabla S1. List of primers

| **Primer** | **Sequence (5´→ 3´)** | **Protocol** | **Size (pb)** |
| --- | --- | --- | --- |
| PActFw  PActRv | TTTTTTGGAGAGGGGGTAAAAAAATGCTGC  TTTTTTCGGCGAACTATATCAGGGCAC | RegCG cloning in  pGM-t | 2001 |
| PActBamEcoFw  PActEcoNotRv | TTTTTTGGATCCGAATTCGGAGAGGGGGTAAAAAAATGCTGC  TCAACGCGGCCGCAAAAAAGAATTCGGCGAACTATATCAGGGCAC | RegCG sub cloning | 2031 |
| CMVcFor01  CMVrev01 | TTTTTTAGATCTGTTGACATTGATTATTGACT  TTTTTTAAGCTTTCGATAAGCCAGTAAGCAGT | CMV promoter  cloning | 646 |
| EnhEcoNotFw:  EnhBglSalRv: | TCGCCGAATTCTTTTTTGCGGCCGCGTTGACATTGATTATTGACT  AAAAAAGTCGACAGATCTCGTCAATGGGGCGGAGTTGTTACGACA | Enhancer CMV cloning | 565 |
| CMVmP1F1  CMVmP1R1 | GATCTGTAACAACTCCGCCCCATTGACGCAAATGGGCGGTAGGCGTGTACGGTGGG  ATAGACCTCCCACCGTACACGCCTACCGCCCATTTGCGTCAATGGGGCGGAGTTGTTACA | Synthesis of Core (figure S1a) | pair 1 |
| CMVmP2F1  CMVmP2R1 | AGGTCTATATAAGCAGAGCTCTCTGGCTAACTAGAGAACCCACTGCTTACTGGCTTATCA  AGCTTGATAAGCCAGTAAGCAGTGGGTTCTCTAGTTAGCCAGAGAGCTCTGCTTAT |  | pair 2 |
| GREp1F  GREp1R  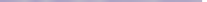 | CGCTAGCAGAACAGGATGTTCTGATCAAAGAGATCCAAAGTCAGAACACGTTG  TGTTCTGACTTTGGATCTCTTTGATCAGAACATCCTGTTCTGCTAGCGAGCT | Synthesis of GRE (figure S1b) | pair 1 |
| GREp2F  GREp2R  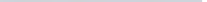 | TTCTAGCTAAAATAACACATTCAGAGAACATGCTGTTCTGATCAAAGAGAT  TTTGATCAGAACAGCATGTTCTCTGAATGTGTTATTTTAGCTAGAACAACG |  | pair 2 |
| GREp3F  GREp3R  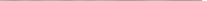 | CCAAAGTCAGAACAAGGTGTTCTAGCTAAAATAACACATTC  TGTTATTTTAGCTAGAACACCTTGTTCTGACTTTGGATCTC |  | pair 3 |
| GREp4F  GREp4R | AGAGAACATGATGTTCTGATCAAAGAGATCCAAAGTCC  TCGAGGACTTTGGATCTCTTTGATCAGAACATCATGTTCTCTGAATG |  | pair 4 |
| InrPActFw01 (F1) InrPActFw02 (F2) InrPActFw03 (F3) INTPActFw01 (F4) INTPActFw02 (F5) INTPActFw03 (F6)  CMVrev03 (R1) | GCCACTGTCGAGTCCGCG CCCGCGAGCACAGGCCTT CACCCGCCACCAGGTAAG GGGTTGCCACTGCGCTTG CGCTTCCTGCTGGGTGTG CGGACCAGCGTTTGCCTC  TATGGGCTATGAACTAATGACC | RT analysis RegCG | 1302  1280  1237  774  573  449 |
| CMVfor (F7)  qLivRev (R2) | CGCAAATGGGCGGTAGGCGTG  TTCGCAGGCGTAGACTTTGT |  | 839 |
| InrPActFw02 (F2) InrPActFw03 (F3) INTPActFw02 (F5)  INTPActFw03 (F6) | CCCGCGAGCACAGGCCTT CACCCGCCACCAGGTAAG CGCTTCCTGCTGGGTGTG  CGGACCAGCGTTTGCCTC | RT analysis ACTB | 651(*s)* /1609(*ns* )  1566(*ns* )  902(*ns* )  778(*ns* ) |
| bactCHOFor01 (F8)  bactCHOrev01 (R3) | GCCAACCGTGAAAAGATGACC  CACGCTCGGTCAGGATCTTC |  | 250 |

Underlined, indicate restriction sites;  *n* , spliced; *ns* , not spliced
