# Supplementary material for: Development of a new promoter to avoid the silencing of genes in the production of recombinant antibodies in chinese hamster ovary cells
Source: J Biol Eng. 2019 Jun 28;13:59. doi: 10.1186/s13036-019-0187-y (PMC6599231; doi:10.1186/s13036-019-0187-y)
Supplement: Supplementary file 2 — Figure S1. Pairing of the primers for the synthesis of a glucocorticoid response element (GRE) tandem. The alignment of the hybridized primers is shown, prior to their ligation with T4 Ligase through the cohesive ends generated. a) Synthesis of short portion of CMV promoter (Core). b) Synthesis of a tandem of five Glucocorticoid Response Element (GRE). In celestial the palindromic elements of the GRE site are shown, and in yellow the cohesive ends compatible with restriction enzymes are shown. (DOCX 13 kb) [file 13036_2019_187_MOESM2_ESM.docx]

**a)**

Pair1 GATCTGTAACAACTCCGCCCCATTGACGCAAATGGGCGGTAGGCGTGTACGGTGGG (CMVmP1F1)

ACATTGTTGAGGCGGGGTAACTGCGTTTACCCGCCATCCGCACATGCCACCCTCCAGATA (CMVmP1R1)

Pair2 AGGTCTATATAAGCAGAGCTCTCTGGCTAACTAGAGAACCCACTGCTTACTGGCTTATCA (CMVmP2F1)

TATTCGTCTCGAGAGACCGATTGATCTCTTGGGTGACGAATGACCGAATAGTTCGA (CMVmP2R1)

**b)**

Pair1: CGCTAGCAGAACAGGATGTTCTGATCAAAGAGATCCAAAGTCAGAACACGTTG (GREp1F)

TCGAGCGATCGTCTTGTCCTACAAGACTAGTTTCTCTAGGTTTCAGTCTTGT (GREp1R)

Pair2: TTCTAGCTAAAATAACACATTCAGAGAACATGCTGTTCTGATCAAAGAGAT (GREp2F)

GCAACAAGATCGATTTTATTGTGTAAGTCTCTTGTACGACAAGACTAGTTT (GREp2R)

Pair3: CCAAAGTCAGAACAAGGTGTTCTAGCTAAAATAACACATTC (GREp3F)

CTCTAGGTTTCAGTCTTGTTCCACAAGATCGATTTTATTGT (GREp3R)

Pair4: AGAGAACATGATGTTCTGATCAAAGAGATCCAAAGTCC (GREp4F)

GTAAGTCTCTTGTACTACAAGACTAGTTTCTCTAGGTTTCAGGAGCT (GREp4R)

Figure S1. **Pairing of the primers for the synthesis of a glucocorticoid response element (GRE) tandem.** The alignment of the hybridized primers is shown, prior to their ligation with T4 Ligase through the cohesive ends generated. **a)** Synthesis of short portion of CMV promoter (Core). **b)** Synthesis of a tandem of five Glucocorticoid Response Element (GRE). In celestial the palindromic elements of the GRE site are shown, and in yellow the cohesive ends compatible with restriction enzymes are shown.
