# Supplementary material for: Development of a new promoter to avoid the silencing of genes in the production of recombinant antibodies in chinese hamster ovary cells
Source: J Biol Eng. 2019 Jun 28;13:59. doi: 10.1186/s13036-019-0187-y (PMC6599231; doi:10.1186/s13036-019-0187-y)
Supplement: Supplementary file 3 — Figure S2. Alignment of promoter sequences of the gene for the beta actin of Gallus (Gall), Human (Hum), Chinese Hamster (CHO), mouse (Mus) and rat (Rat). In Amarillo, the CAAT, TATA and CArG boxes stand out. In green, the XhoI restriction site and the start codon of beta-actin are highlighted. The arrows> and < indicate the start and end, respectively of the CpG Island, the start of transcription and the intron I for the sequence of the CHO cell genome. (DOCX 16 kb) [file 13036_2019_187_MOESM3_ESM.docx]

Start CpG island (CHO)>

Gall ccccaattttgtatttatttattttttaattattttgtgcagcgatgggggcgggggggg -1312

Hum cctcctcttcctcaatctcgctctcgctctttttttttttcgcaaaaggaggggagaggg -997

CHO ------tttttttttttttttttttttttttttttttttttgcaaaaggaggggagaggg -1180

Mus -----------tttttttcttcttctttttttttttttttttcaaaaggaggggagaggg -1242

Rat ------------------tttttttttttttttttttttttgcaaaaggaggggagaggg -1165

* ** **** * * * ** * ** * ***

Gall ggggggcgcgcgccaggcggggcggggcggggcgaggggcggggcggggcgaggcggaga -1252

Hum ggtaaaaaaatgctgcact-------gtgcggcgaagcc--------------ggtgagt -1049

CHO ggtaaaaaaatgctgcact-------gtgcggctaggcc--------------ggtgagt -1141

Mus ggtaaaaaaatgctgcact-------gtgcggcgaggcc--------------ggtgagt -1203

Rat ggtaaaaaaatgctgcact-------gtgcggcgaggcc--------------ggtgagt -1126

** ** * * * *** * * * ***

CAT CArG XhoI

Gall ggtgcggcggcagccaatcagagcggcgcgctccgaaagtttccttttatggcgaggcgg -1192

Hum gagcggcgcggggccaatcagcgtgcgccgttccgaaagttgccttttatggctcgagcg -989

CHO gagcggcgcggagccaatcagcgctcgccgttccgaaagttgccttttatggctcgagtg -1081

Mus gagcgacgcggagccaatcagcgcccgccgttccgaaagttgccttttatggctcgagtg -1143

Rat gagcgacgcggagccaatcagcgcccgccgttccgaaagttgccttttatggctcgagtg -1066

* * ********* * ** ********** *********** * *

>Start mRNA (CHO)

TATA

Gall cggcggcggcggccctataaaaagcgaagcgcgcggcgggcgggagtcgctgcgttgcct -1132

Hum gccgcggcggcgccctataaaacccagcggcgcgacgcgccaccaccgccgagaccgcgt -929

CHO gccgctgtggcgtcctataaaacccggcggcgcaacgcgcagccactgtcgagtccgcgt -1021

Mus gccgctgtggcgtcctataaaacccggcggcgcaacgcgcagccactgtcgagtcg-cgt -1084

Rat gccgctgtggcgtcctataaaacccggcggcgcaacgcgcagccactgtcgagtccgcgt -1006

* * ********* * * * * * * *

Gall tcgccccgtgccccgct--ccg-----------------cgccgcctc------------ -1103

Hum ccgccccgcgagcacagagcctcgcctttgccgatccgccgcccgtccacacccgccgcc -869

CHO ccacccgcgagcacaggcctttcgcag---ctctttcttcgccgctccacacccgccacc -964

Mus ccacccgcgagcacagcttctttgcag---ctccttcgttgccggtccacacccgccacc -1027

Rat ccacccgcgagtacaaccttcttgcag---ctcctccgtcgccggtccacacccgccacc -949

* *** *** *

>Start intron (CHO)

Gall -------------------gcgccgcccgccccggctctgactgaccgcgttact----- -1067

Hum aggtaagcccggccagccgaccggggcaggcggctcacggcccggccgcaggcggccgcg -809

CHO aggtaagcagggacaacaggcccagccggccacagccctcccgtgggcagtgaccgcgct -904

Mus aggtaagcagggacgccgggcccagcgggccttcgctctctcgtggcta-gtacctcact -968

Rat aggtaagcagggacgtcgggcccagcgggccccaactttaccttggcca-ctacctcgct -890

* * * * * *

////////////////////////////////////////////////////////////

CArG

Gall cgagccgcagccattgccttttatggtaatcgtgcgagagggcgcagggacttcctttgt -419

Hum ctccgaccagtgtttgccttttatggtaataacgcggccggcccgg---cttcctttgtc -117

CHO tccggaccagcgtttgcctcttatggtaataacgcggccggcctgg---gcttcctttgt -133

Mus tccgggccagcgtttgccttttatggtaataatgcggccggtctgc---gcttcctttgt -131

Rat tccgggccagcgtttgccttttatggtaataatgcggctgtcctgc---gcttcctttgt -131

*** ****** ********** *** * * * * **

////////////////////////////////////////////////////////////

End intron (CHO)< Star Codon

Gall gaccggcggggtttatatcttcccttctctgttcctccgcagccagccatggatgatgat 12

Hum -------------------agcgcgcccggctattctcgcagctcaccatggatgatgat 12

CHO ggcccgccagtgaccgcgaccctcttttgtgccctgatatagttcgccatggatgacgat 12

Mus ggccccgaggtgactatagccttcttttgtgtct--tgatagttcgccatggatgacgat 12

Rat ggctccgcggtgaccatagccctcttttgtgcct--tgatagttcgccatggatgacgat 12

** ** ********** ***

////////////////////////////////////////////////////////////

En CpG Island (CHO)<

Gall atgggttgagtacgcagcctccgcggagcatcctgtgttggagcagttgctcagtccctt 320

Hum -----------tcccctccatcgtggggcgccccaggcacca--ggtaggg—gagctgg 136

CHO -----------tcccatccatcgtgggccgccctaggcacca--ggtaggt—gaccctt 136

Mus -----------tcccctccatcgtgggccgccctaggcacca--ggtaagt—gacctgt 136

Rat -----------tcccctccatcgtgggccgccctaggcacca--ggtaagt—gaccctt 136

* * ** ** ** * ** * * ** * *

**Figure S2.** Alignment of promoter sequences of the gene for the beta actin of Gallus (Gall), Human (Hum), Chinese Hamster (CHO), mouse (Mus) and rat (Rat). In Amarillo, the CAAT, TATA and CArG boxes stand out. In green, the *Xho*I restriction site and the start codon of beta-actin are highlighted. The arrows> and <indicate the start and end, respectively of the CpG Island, the start of transcription and the intron I for the sequence of the CHO cell genome.
